# Identification and verification of IGFBP3 and YTHDC1 as biomarkers associated with immune infiltration and mitophagy in hypertrophic cardiomyopathy

**DOI:** 10.3389/fgene.2022.986995

**Published:** 2022-10-04

**Authors:** Yao Li, Wei Zhang, Yan Dai, Keping Chen

**Affiliations:** ^1^ State Key Laboratory of Cardiovascular Disease, Fuwai Hospital, National Center for Cardiovascular Diseases, Chinese Academy of Medical Sciences and Peking Union Medical College, Beijing, China; ^2^ Department of Urology, Beijing Hospital, National Center of Gerontology, Institute of Geriatric Medicine, Chinese Academy of Medical Sciences, Beijing, China

**Keywords:** hypertrophic cardiomyopathy, N6-methyladenosine methylation modification, immune infiltration, mitophagy, energy metabolism

## Abstract

**Background:** Hypertrophic cardiomyopathy (HCM) is the main cause of sudden cardiac death among young adults, yet its pathogenesis remains vague. N6-methyladenosine (m6A) methylation modification was involved in various cardiovascular diseases such as coronary heart disease and heart failure, although its influence on HCM remains unclear. This study aimed to explore the potential role of m6A in the diagnosis and pathogenesis of HCM.

**Methods:** GSE36961 including 106 HCM and 39 controls was used in the study. The HCM-related m6A regulators were selected using support vector machine recursive feature elimination and random forest algorithm. A significant gene signature was then established using least absolute shrinkage and selection operator and then verified by GSE130036. Subgroup classification of HCM was performed based on the expression of m6A biomarkers. Gene set variation analysis was employed to explore the functional difference between distinct subgroups. Weighted gene co-expression network analysis was used to determine the m6A-related hub module. Single-sample gene set enrichment analysis was conducted to assess the immune and mitophagy features between subgroups. Besides, transfection of recombinant plasmids with targeted genes into H9c2 cells was performed to further verify the function of the significant biomarkers.

**Results:** Significant difference existed in m6A landscape between HCM and control patients, among which IGFBP3 and YTHDC1 were identified as the independent biomarkers of HCM. Highly infiltrated immune cells (MDSC, macrophages, etc.), more enriched immune-related pathways (TNFα signaling *via* NFκB and IL6-JAK-STAT3 signaling) and cardiac remodeling-associated pathways (epithelial mesenchymal transition, angiogenesis, etc.) were identified in the subgroup with higher IGFBP3. Consistently, overexpression of IGFBP3 in H9c2 cells led to upregulation of extracellular-matrix-related genes (COL1A2, COL3A1 and MMP9) and inflammation-related genes (TNFα and IL6). Besides, higher YTHDC1 expression seemed to be consistent with less-activated mitophagy (PINK1-PRKN mediated mitophagy) and energy metabolism. Further experiments demonstrated that overexpression of YTHDC1 resulted in up-regulation of PINK and PRKN in cardiomyocytes, which are essential genes mediating mitophagy.

**Conclusion:** Two m6A readers **(**IGFBP3 and YTHDC1) well distinguished HCM and may facilitate clinical diagnosis. IGFBP3 may play a role in the immune-microenvironments and remodeling of cardiac tissues, while YTHDC1 may influence mitophagy and energy metabolism in HCM.

## Introduction

Hypertrophic cardiomyopathy (HCM), a hereditary heart disease characterized by asymmetric cardiac hypertrophy, has become the main cause of sudden cardiac death among adolescents and young adults (Semsarian et al., 2015; Marian and Braunwald, 2017). Mutations in the genes encoding sarcomere-associated proteins are generally considered the main cause of HCM, with MYH7 and MYBPC3 the two most common genes involved (Marian and Braunwald, 2017). However, gene mutations could not tell the whole story of HCM since these mutations were not always consistent with clinical phenotypes (Marian and Braunwald, 2017), and there is still a large gap on how these mutations result in clinical manifestations. The pathogenesis of HCM remains to be further clarified.

RNA modification, an essential form of epigenetics, was known to mediate numerous biological processes through regulating RNA processing and metabolism (Roundtree et al., 2017a). N6-methyladenosine (m6A) methylation is the most abundant type of RNA modification, mediated by methyltransferases (writers), demethylases (erasers) and binding proteins (readers) (Frye et al., 2018; Yang et al., 2018). M6A methylation modification has been reported to play a key role not only in cancers (Li et al., 2021) and immune-related conditions (Dai et al., 2021; Zhang et al., 2021; Zhao et al., 2021), but also in various cardiovascular diseases including coronary heart disease, hypertension, and heart failure (Wu et al., 2020). However, whether m6A regulators play a role in the pathogenesis of HCM remains unclear.

**FIGURE 1:**
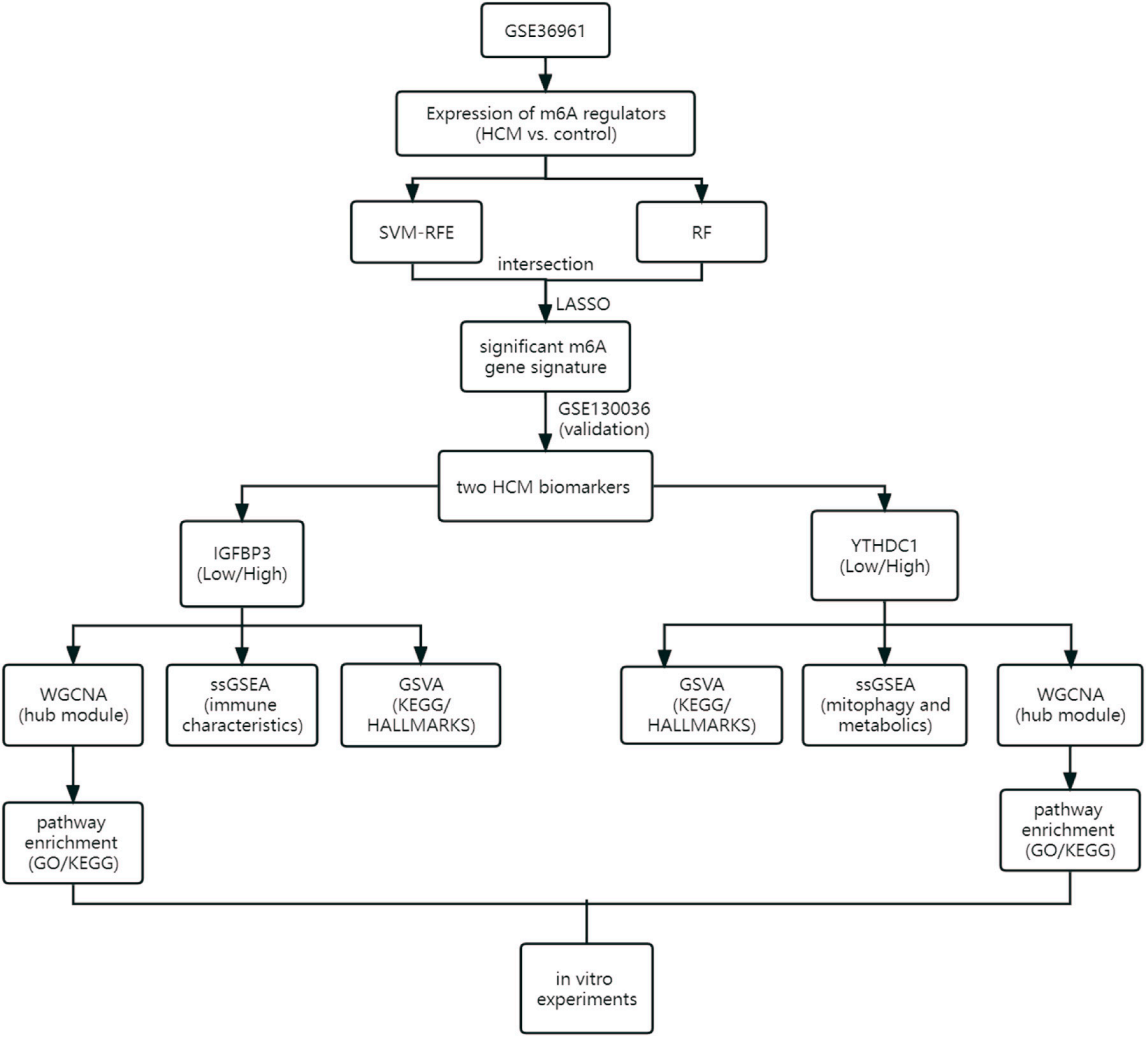
Study flow diagram. GO, Gene Ontology; GSVA, gene set variation analysis; HCM, hypertrophic cardiomyopathy; KEGG, Kyoto Encyclopedia of Genes and Genomes; LASSO, least absolute shrinkage and selection operator; RF, random forest; ssGSEA, single sample gene set enrichment analysis; SVM-RFE, support vector machine recursive feature elimination; WGCNA, weighted gene co-expression network analysis.

As the flowchart showed in Figure 1, this study aimed to explore the potential role of m6A methylation modification in the diagnosis and pathogenesis of HCM. Datasets from GEO database were downloaded and analyzed through bioinformatical algorithms. The potential m6A biomarkers for HCM were firstly explored. Subgroup classification of HCM was then performed based on the expression of significant m6A biomarkers, and difference in biological function was assessed between the distinct m6A patterns. Besides, *in vitro* experiments were employed to further testify the role of significant m6A regulators in cardiomyocytes.

## Materials and methods

### Data collection and preprocessing

Gene expression profile data were obtained from the Gene Expression Omnibus (GEO) database (https://www.ncbi.nlm.nih.gov/geo). The inclusion criteria were as follows: 1) Microarray or high-throughput sequencing data including both HCM and control samples; 2) adequate sample size for analysis; 3) the specimens were human cardiac tissues. Datasets with candidates in any clinical trials involving drugs or interventions should be excluded. GSE36961 (106 patients with HCM and 39 controls) (Bos et al., 2020) and GSE130036 (28 HCM patients and nine healthy donors) (Liu et al., 2019) were finally used in the current study. Data from the two datasets were normalized by the “limma” package (Robinson et al., 2010) in R software version 4.2.0 (https://www.r-project.org/). Datasets from the GEO database are free of charge and in no need for ethical approval. Figure 1 illustrates an overview of the study, and all the R scripts used are available at https://www.jianguoyun.com/p/DRg0Rt0QuZ_fChjem8sEIAA.

### Identification of certain m6A regulators as hypertrophic cardiomyopathy biomarkers

The m6A RNA methylation regulators were obtained from previous publications (Zaccara et al., 2019; Zhou et al., 2022), including 9 writers (METTL3, METTL14, METTL16, WTAP, VIRMA, ZC3H13, RBM15, RBM15B, CBLL1), 15 readers (YTHDC1, YTHDC2, YTHDF1, YTHDF2, YTHDF3, HNRNPC, FMR1, LRPPRC, HNRNPA2B1, IGFBP1, IGFBP2, IGFBP3, RBMX, ELAVL1, IGF2BP1) and two erasers (FTO and ALKBH5). The chromosomal positions of the m6A regulators identified in cardiac tissues were illustrated using the “RCircos” package (Dai et al., 2021). Spearman correlation was used to assess the relationship between m6A regulators. The R package “limma” was employed to identify the differentially expressed m6A regulators between HCM and control groups, which were then visualized through “ggplot2”. Of note, Wilcox rank sum test was used for differential analysis and *p* < 0.05 was considered statistically significant.

Significant m6A regulators were selected through random forest (RF) model with “randomForest” package (Breiman, 2001) and support vector machine recursive feature elimination (SVM-RFE) method with e1071 package (Noble, 2006). Significant gene signature was then established by least absolute shrinkage and selection operator (LASSO) algorithm with glmnet R package (Vasquez et al., 2016). The gene signature was then evaluated by receiver operating curves (ROC). Next, the external validation dataset GSE130036 was used to verify the gene signature determined in GSE36961. Validated m6A regulators were finally reckoned as the significant biomarkers for HCM.

### Functional analyses of the two m6A biomarkers

HCM patients from GSE36961 were divided into two subgroups based on the median expression of IGFBP3 and YTHDC1, respectively. The gene set variation analysis (GSVA) algorithm was conducted to demonstrate the differentially activated pathways between the distinct m6A subgroups through the R package “GSVA” (Hänzelmann et al., 2013) with “h.all.v7.5.1. symbols” and “c2. cp.kegg.v7.5.1. symbols” downloaded from MSigDB database (https://www.gsea-msigdb.org/gsea/msigdb/index.jsp). The difference in pathway activation score was evaluated by R package “limma” with adjusted *p* < 0.05 as the cut-off criterion (Ritchie et al., 2015; Zhang et al., 2021).

Weighted gene co-expression network analysis (WGCNA) was used to explore m6A subgroup-related gene modules *via* the WGCNA R package (Langfelder and Horvath, 2008). The relationship between various modules and subgroups was evaluated through Spearman correlation analysis. The most significant hub module was then enriched in Gene Ontology (GO) and Kyoto Encyclopedia of Genes and Genomes (KEGG) pathways using the “clusterProfiler” package (Wu et al., 2021).

Immune-associated gene sets were downloaded from the ImmPort database (Bhattacharya et al., 2014) and single-sample gene-set enrichment analysis (ssGSEA) was then performed to assess the different immune characteristics between distinct IGFBP3 subgroups. Besides, “Estimate” R package was employed to calculate the immune score of HCM patients (Yoshihara et al., 2013). The difference between subgroups was analyzed through Wilcox rank sum test and *p* < 0.05 was considered statistically significant. The differential analysis was then visualized through “ggplot2”. Spearman correlation was used to assess the relationship between IGFBP3 and immune cells.

Mitophagy-related gene sets [PINK1-PRKN mediated mitophagy (R-HSA-5205685.3) and receptor-mediated mitophagy (R-HSA-8934903.3)] and metabolic-related gene sets [glycolysis (R-HSA-70171.6), mitochondrial beta-oxidation of saturated fatty acids (R-HSA-77286.2), mitochondrial beta-oxidation of unsaturated fatty acids (R-HSA-77288.2), propionyl-CoA catabolism (R-HSA-71032.2), pyruvate metabolism and tricarboxylic acid (TCA) cycle (R-HSA-71406.1), respiratory electron transport and ATP synthesis (R-HSA-163200.1)] were downloaded from Reactome database (https://reactome.org/). The ssGSEA and Wilcox rank sum test were then performed to assess the different mitophagy and metabolic characteristics between distinct YTHDC1 subgroups and then visualized using “ggplot2”, with *p* < 0.05 the significance criteria.

### *In vitro* experiments to explore the role of the two m6A biomarkers in cardiomyocytes

Cell culture. The H9c2 (cl-0089) cell lines were purchased from the Procell Life Science &Technology Co., Ltd. (China). Cells were cultured in Dulbecco’s Modified Eagle Medium (Gibco, United States) added with 10% fetal bovine serum (FBS) and 1% penicillin/streptomycin (Gibco, United States) at 37°C with 5% CO2. Passage was performed when cells grew to 80% confluence.

Plasmid construction and cell transfection. The overexpression of IGFBP3 and YTHDC1 was achieved by the construction of pcDNA3.1-IGFBP3 and pcDNA3.1-YTHDC1. Besides, pcDNA3.1-NC was constructed as control plasmids. Recombinant plasmids were then transfected into H9c2 cells when 60%–70% confluence was reached according to the Lipofectamine 2000 reagent (Invitrogen, United States) instructions. The medium was changed 6 hours after the transfection.

The real-time quantitative reverse transcription polymerase chain reaction (RT-qPCR) was performed 48–72 h after the transfection. RNA was extracted using TRIzol Reagent (Invitrogen, United States). The cDNA was synthesized with HiScript II Q RT SuperMix (Vazyme, China). RT-qPCR was performed according to the instructions of the 2×RealStar Fast SYBR qPCR Mix kit (GenStar, China). The relative mRNA levels of target genes were determined according to the 2-deltadeltact method with GAPDH as an internal reference. The independent-sample *t*-test was then employed to assess the differential expression of the targeted genes between overexpression groups and control groups with *p* < 0.05 the significance criteria. The primer sets of targeted genes were illustrated in Supplementary Table S1.

### Additional methods and statistics

Wilcox rank sum test (non-normal distribution) and independent-sample *t*-test (normal distribution) were employed for differential analyses. Shapiro-Wilk was used for normality test. General criterion of statistical significance was *p* < 0.05. Of note, the identification of significant pathways or biological processes using GSVA, GO and KEGG analyses, which always involves multiple testing, was subject to correction with false discovery rate (FDR)-adjusted methods, and adjusted *p* < 0.05 was considered significant for these analyses. All the above analyses were performed using SPSS 23.0 and R software (version 4.2.0). GraphPad Prism (version 8) and Adobe Illustrator also helped in graphing.

## Results

### The landscape of m6A regulators in hypertrophic cardiomyopathy patients

**FIGURE 2:**
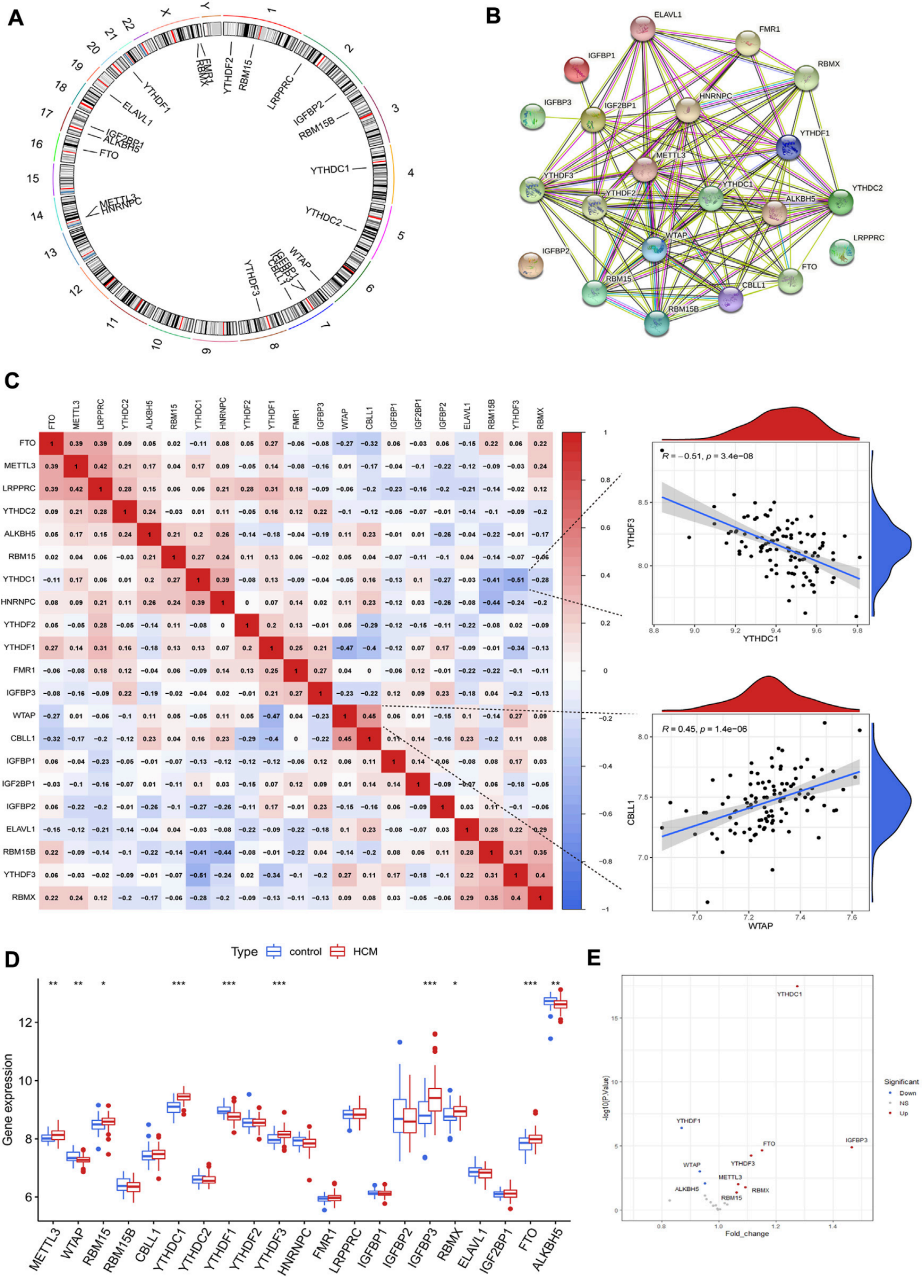
The landscape of m6A regulators in hypertrophic cardiomyopathy (HCM) patients. **(A)** Chromosomal positions of the 21 m6A regulators identified in cardiac tissues of HCM. **(B)** Protein-protein interaction network of the m6A regulators. **(C)** Spearman correlation analysis between the 21 m6A regulators in HCM and the scatterplots showing the two pairs of m6A regulators with the highest positive and negative correlation. **(D)** Box plot demonstrating the differential expression of 21 m6A regulators between HCM and non-HCM using Wilcox rank sum test. **(E)** Volcano plot showing a summary of the 10 differentially expressed m6A regulators. *p < 0.05, **p < 0.01, ***p < 0.001.

A total of 21 m6A regulators were finally identified in cardiac tissues from GSE36961, including 5 writers (METTL3, WTAP, RBM15, RBM15B and CBLL1), 14 readers (YTHDC1, YTHDC2, YTHDF1, YTHDF2, YTHDF3, HNRNPC, RMR1, LRPPRC, IGFBP1, IGFBP2, IGFBP3, RBMX, ELAVL1 and IGF2BP1) and two erasers (FTO and ALKBH5). The chromosomal positions of the detected m6A regulators were shown in Figure 2A. These m6A regulators had a quite close interaction with each other as shown in the protein-protein interaction network (Figure 2B). Correlation analysis further demonstrated the close relationship between m6A regulators at mRNA level, with WTAP and CBLL1 the most positively correlated ones while YTHDC1 and YTHDF3 the most negatively correlated ones (Figure 2C). Ten m6A regulators were differentially expressed between HCM and control groups as shown in Figures 2D,E. YTHDF1, ALKBH5 and WTAP were downregulated in HCM patients while the other 7 m6A regulators (including YTHDC1 and IGFBP3) were upregulated. Besides, IGFBP3 had the largest increase in fold change whereas YTHDC1 was the most statistically different m6A regulator.

### Identification of m6A regulator as hypertrophic cardiomyopathy biomarkers

**FIGURE 3:**
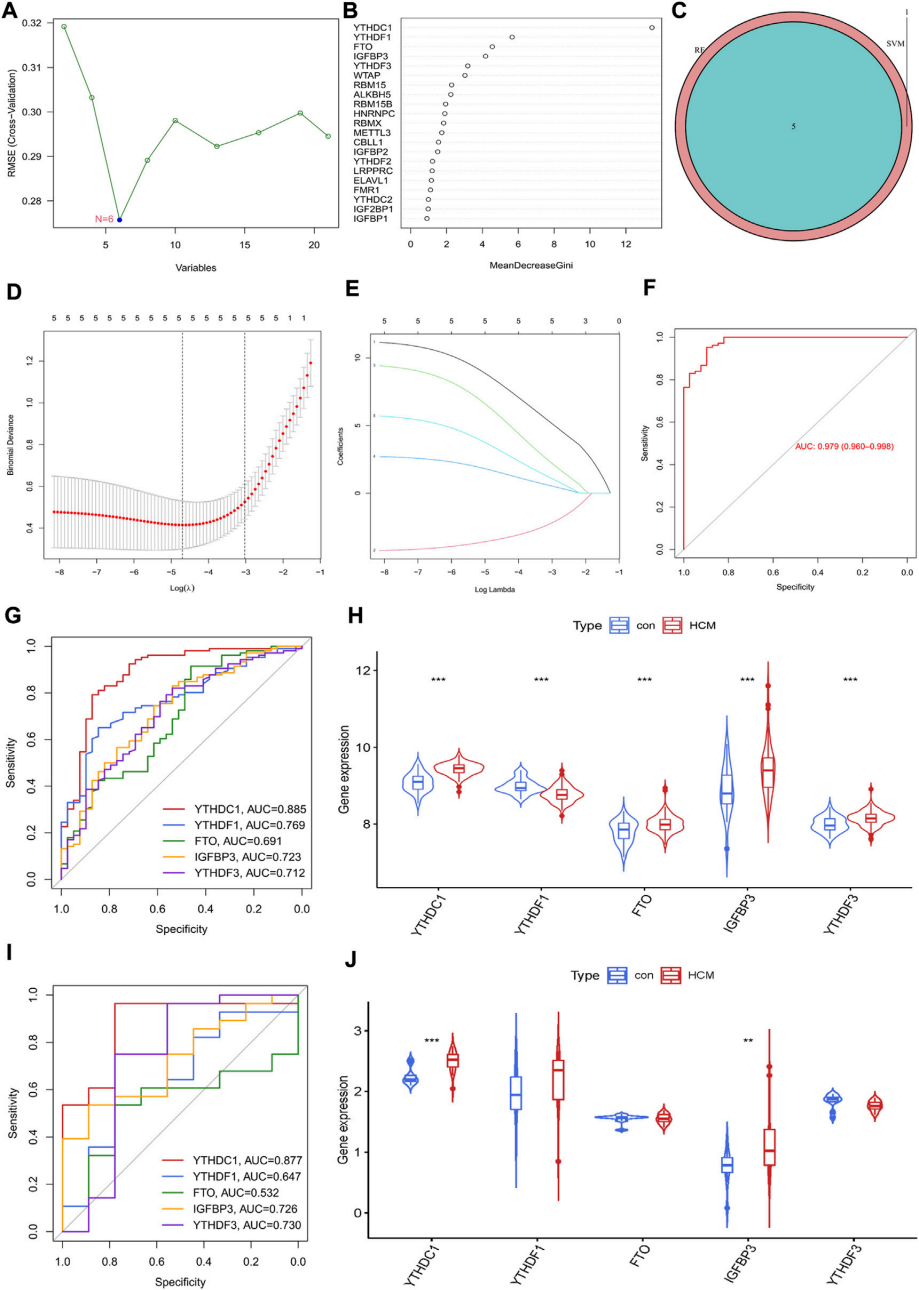
Identification of m6A Regulator as biomarkers of hypertrophic cardiomyopathy. **(A)** Selection of significant m6A regulators by support vector machine recursive feature elimination (SVM-RFE) model. **(B)** Selection of significant m6A regulators by random forest (RF) model. **(C)** Venn diagram of five overlapped candidate genes shared by the SVM-RFE and RF algorithms. **(D,E)** Least absolute shrinkage and selection operator (LASSO) coefficient profiles of hub genes and cross-validation of the model. **(F)** receiver operating curves (ROC) of LASSO model in GSE36961. **(G,H)** ROC assessment and difference analysis (Wilcox rank sum test) of the candidate biomarkers using GSE36961. **(I)** Validation of the diagnostic value of candidate biomarkers using GSE130036. **(J)** The expressions of candidate diagnostic biomarkers in the GSE130036 dataset (Wilcox rank sum test). *p < 0.05, **p < 0.01, ***p < 0.001.

The potential diagnostic value of m6A regulators for HCM was further explored. An SVM-RFE model was firstly established and six significant m6A regulators were identified (Figure 3A). Meanwhile, the first five most important genes from the RF model were determined (Figure 3B). Five genes were obtained by overlapping the significant m6A regulators from the above two models (Figure 3C). LASSO was then employed and a robust m6A gene signature including YTHDC1, YTHDF1, FTO, IGFBP3 and YTHDF3 was finally established (Figures 3D,E). The diagnostic value of the gene signature was assessed by ROC curve with an AUC of 0.979 (95% CI, 0.960–0.968) (Figure 3F). The diagnostic value of every single gene in the signature was also evaluated and YTHDC1 had the largest AUC of 0.885; all genes were significantly increased in HCM group except for YTHDF1 (Figures 3G,H). GSE130036 was employed to verify the results from GSE36961 (Figures 3I,J). YTHDC1 and IGFBP3 were also significantly increased in HCM group with an AUC of 0.877 and 0.726 respectively, while the expression of the other three regulators showed no statistical significance between groups. Therefore, YTHDC1 and IGFBP3 were considered as the significant biomarkers of HCM.

### The potential role of IGFBP3 in the pathogenesis of hypertrophic cardiomyopathy

**FIGURE 4:**
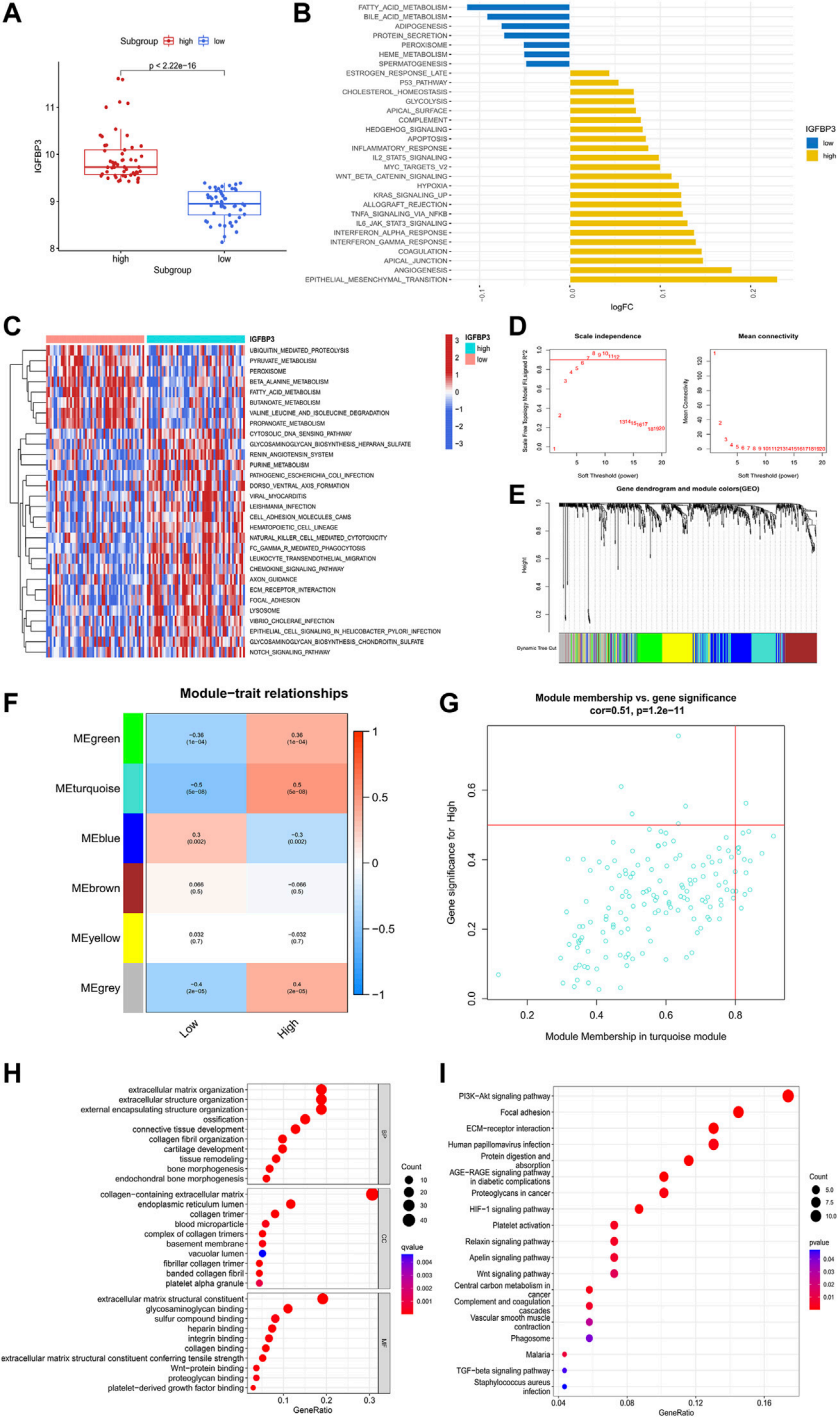
Biological enrichment analysis of the two m6A subgroups based on IGFBP3 expression. **(A)** Difference in the expression of IGFBP3 between subgroups (Wilcox rank sum test). **(B)** The top 30 different HALLMARKS pathways between distinct m6A subgroups. **(C)** The top 30 different Kyoto Encyclopedia of Genes and Genomes pathways between distinct m6A subgroups. **(D)**The process of weighted gene co-expression network analysis (WGCNA). Identification of the soft-thresholding power for the optimal scale-free topology fit index (left) and mean connectivity (right). The optimal soft threshold power was six. **(E)** The process of WGCNA. The dendrogram represented the gene clustering, and the color row beneath indicated the six modules identified after dynamic tree cut and mergence of similar modules. **(F)** Module-trait relationships demonstrating correlation between the modules identified through WGCNA and IGFBP3 subgroups using spearman analysis. **(G)** A scatterplot of gene significance (GS) vs. module membership (MM) in turquoise module. **(H)** Gene Ontology analysis of turquoise module illustrated by bubble plot. **(I)** Kyoto Encyclopedia of Genes and Genomes analysis of turquoise module illustrated by bubble plot.

To investigate the potential role of IGFBP3 in the pathogenesis of HCM, two distinct subgroups were identified based on IGFBP3 expression, with 53 patients in each subgroup (Figure 4A). Pathway enrichment analysis was performed to uncover the different biological features between groups. A total of 30 significantly different HALLMARKS pathways were identified (Figure 4B). The epithelial mesenchymal transition, angiogenesis, and inflammation-related pathways (including interferon response pathways, IL6-JAK-STAT3 signaling, TNFα signaling *via* NFκB) were highly enriched in the subgroup with high IGFBP3 expression, while fatty acid metabolism, bile acid metabolism and adipogenesis pathways were more enriched in the subgroup with low IGFBP3. Among 60 different KEGG pathways, renin angiotensin system, natural killer cell mediated cytotoxicity, leukocyte trans-endothelial migration, chemokine signaling, and extracellular matrix (ECM) receptor interaction were more enriched in subgroup with high IGFBP3, while the counterpart subgroup had highly enriched fatty acid metabolism and β-alanine metabolism pathways (Figure 3C). The GSVA results indicated that pathways involving cardiac remodeling and inflammation response were highly enriched in the subgroup with high IGFBP3 expression.

WGCNA was then conducted to get the hub module associated with IGFBP3 subgroup (Figures 4D–G). The optimal soft threshold power was 6 with the cutoff R^2^ equal to 0.90. Six modules were finally established with the minimum genes of 50 per module and a cutoff height of 0.25 when merging similar modules. According to the module-trait relationship, the turquoise module was most positively correlated to high IGFBP3 expression (Cor = 0.5, *p* < 0.001). The biological function of the hub module was explored through GO and KEGG analysis (Figures 4H,I). The top 10 pathways in BP were mainly associated with the organization of ECM and collogen. PI3K-Akt signaling pathway, focal adhesion, ECM-receptor interaction were the top three pathways in KEGG. In a word, IGFBP3-related module was significantly associated with cardiac ECM remodeling.

**FIGURE 5:**
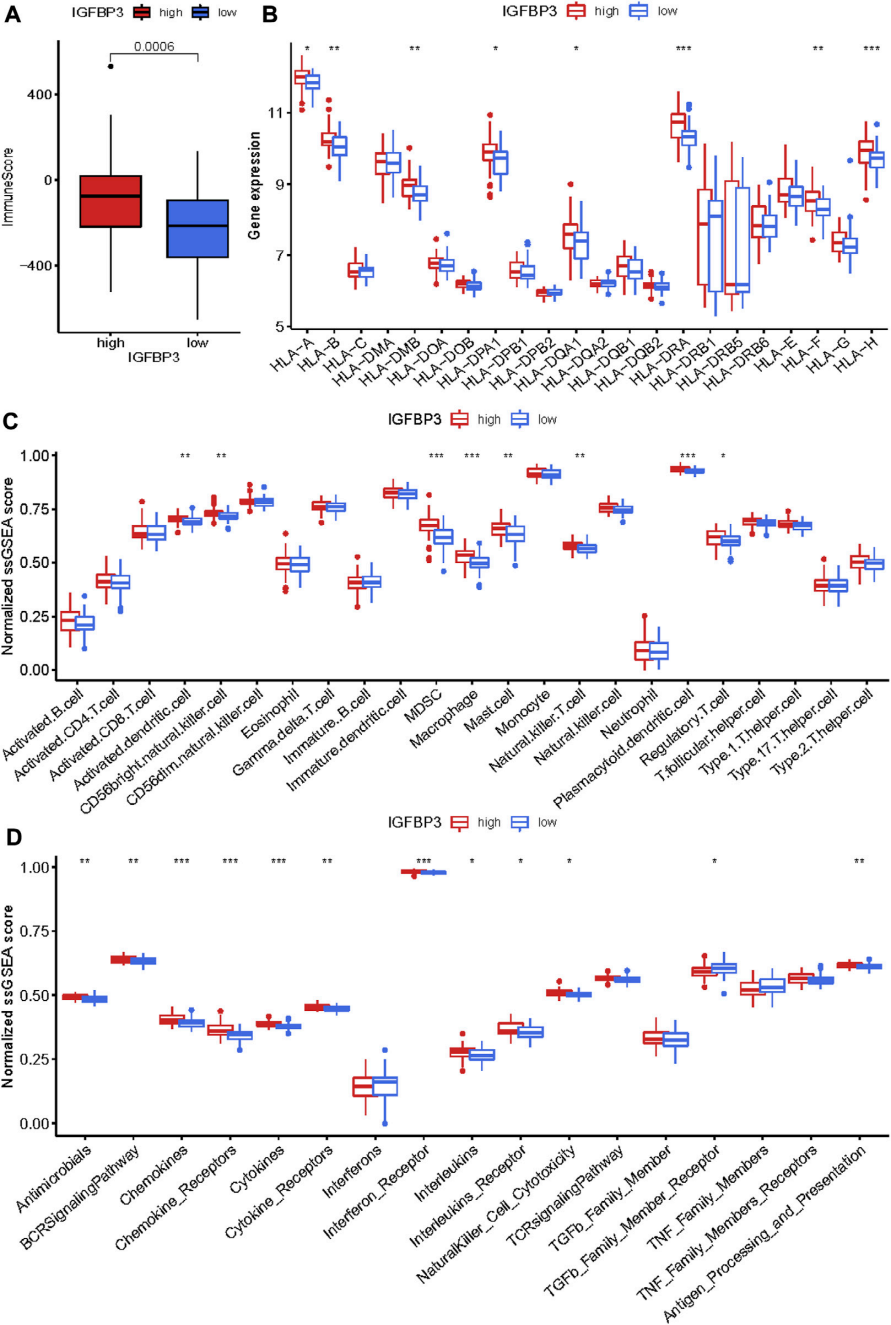
Immune infiltration difference between the two m6A subgroups based on IGFBP3 expression using Wilcox rank sum test. **(A)** Different immune scores between the two subgroups. **(B)** Different expression of human leukocyte antigen (HLA) genes. **(C)** Difference in the abundance of immune cells between the two subgroups. **(D)** Difference in the activation of immune responses. *p < 0.05, **p < 0.01, ***p < 0.001.

**FIGURE 6:**
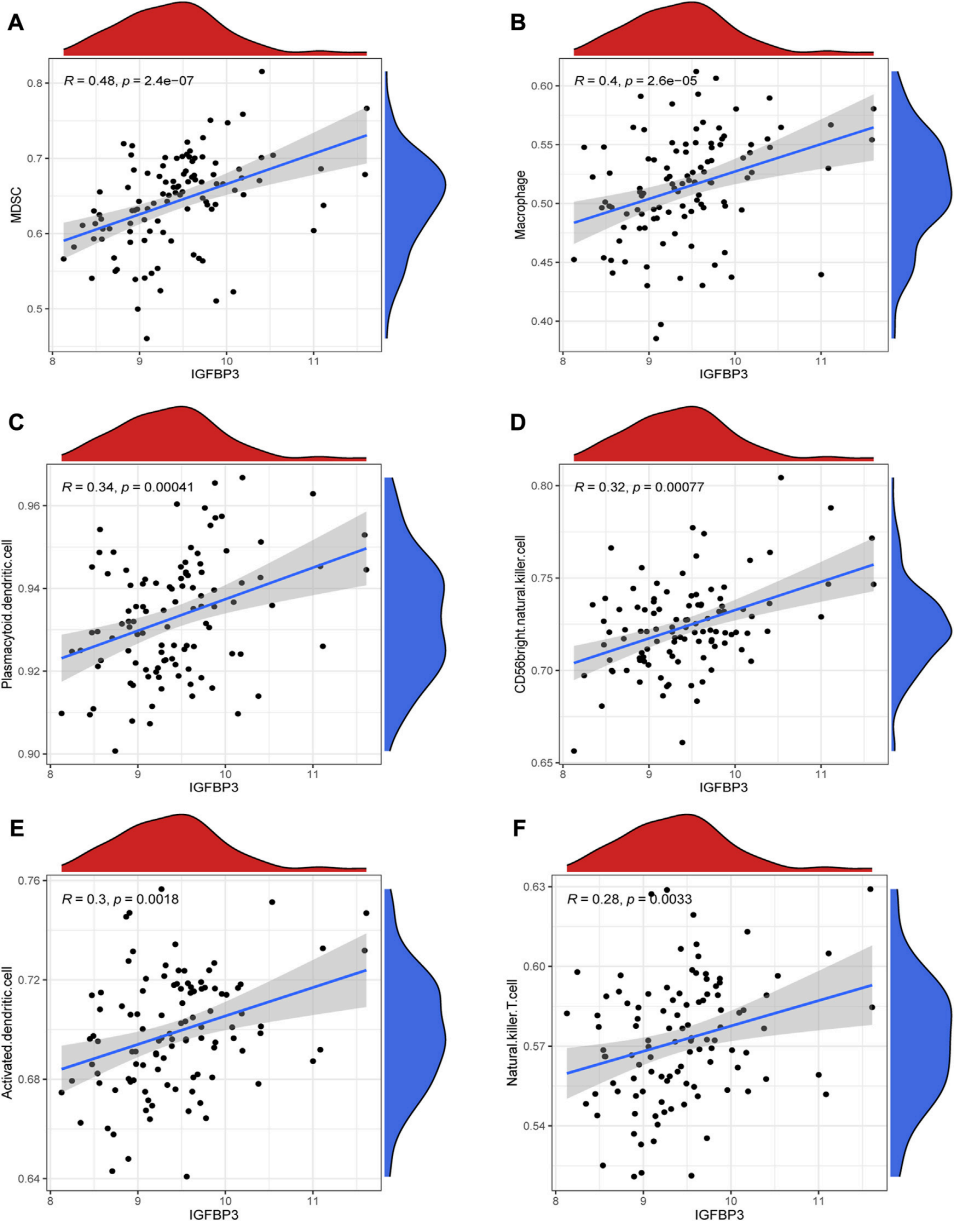
Correlation between IGFBP3 and immune cells in hypertrophic myocardiopathy (HCM) using spearman correlation analysis. **(A)** Correlation between IGFBP3 and MDSC. **(B)** Correlation between IGFBP3 and macrophage. **(C)** Correlation between IGFBP3 with plasmacytoid dendritic cell. **(D)** Correlation between IGFBP3 with CD56 bright natural killer cell. **(E)** Correlation between IGFBP3 with activated dendritic cell. **(F)** Correlation between IGFBP3 with natural killer T cell.

Given that higher IGFBP3 expression was consistent with more enriched inflammation-associated pathways in GSVA, the comparison of immune characteristics between the two subgroups was further performed. The subgroup with high IGFBP3 had higher immune score (Figure 5A), and further detailed comparison in immune cell infiltration, immune response, and human leukocyte antigen (HLA) expression demonstrated similar trends (Figures 5B–D). High IGFBP3 subgroup had higher abundance of infiltrated activated dendritic cell, CD56 bright natural killer cell, MDSC, macrophage, mast cell, natural killer T cell, plasmacytoid dendritic cell, and regulatory T cell. For immune response, 11 immune-activated pathways (interleukins, cytokines, interferon receptor, etc.) were more enriched in the subgroup with high IGFBP3. Only TGFβ family member receptor signaling, whose biological function was associated with immunosuppression, was less activated. All the eight differentially expressed HLA genes showed higher expression level in high IGFBP3 subgroup, such as HLA-DRA, HLA-DMB, and HLA-H. Besides, correlation analysis showed that IGFBP3 was positively associated with the above mentioned differentially infiltrated immune cells in HCM, and the top six pairs with high correlation coefficients and *p* < 0.05 were illustrated in Figure 6. These results indicated that IGFBP3 may play a role in the immune-microenvironments of HCM cardiac tissue.

### The potential role of YTHDC1 in the pathogenesis of hypertrophic cardiomyopathy

**FIGURE 7:**
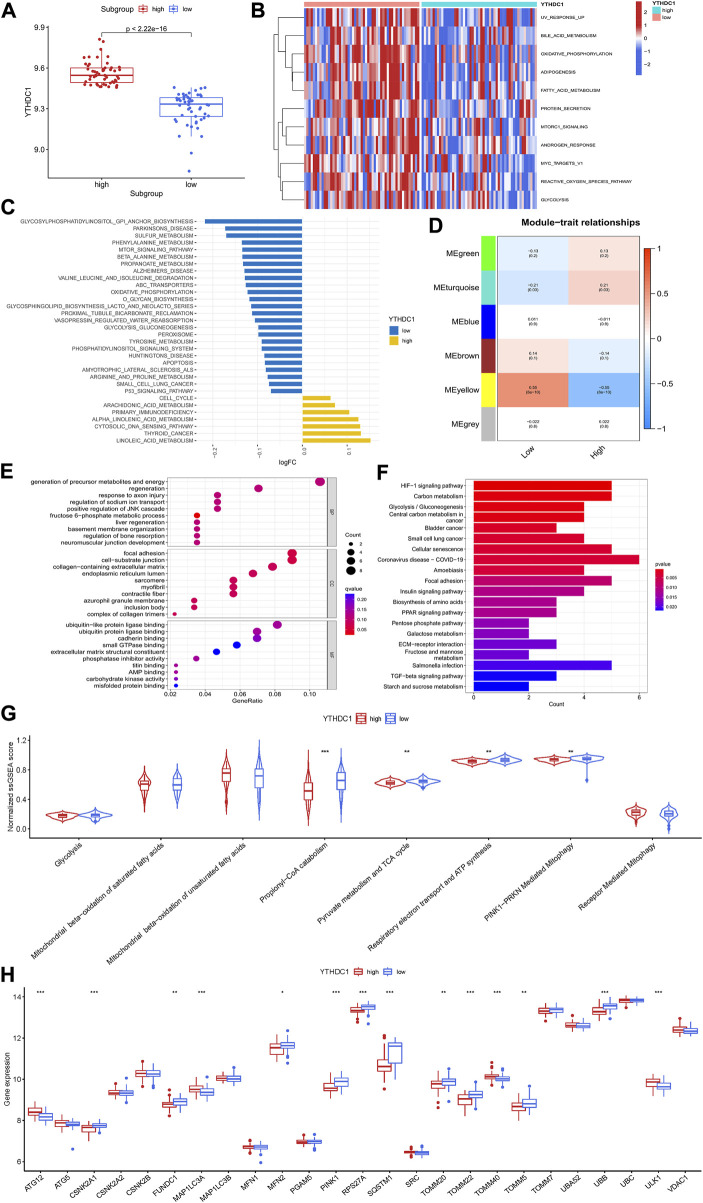
Biological enrichment analysis of the two m6A subgroups based on YTHDC1 expression. **(A)** Difference in the expression of YTHDC1 between subgroups (Wilcox rank sum test). **(B)** Different HALLMARKS pathways between distinct subgroups. **(C)** Different Kyoto Encyclopedia of Genes and Genomes pathways between distinct subgroups. **(D)** Correlation between the modules from weighted gene co-expression network analysis and YTHDC1 subgroups using spearman analysis. **(E)** Gene Ontology analysis of yellow module illustrated by bubble plot. **(F)** Kyoto Encyclopedia of Genes and Genomes analysis of yellow module illustrated by histogram. **(G)** Violin plot showing the difference in energy metabolism and mitophagy-related pathways between the two subgroups (Wilcox rank sum test). **(H)** Difference in the expression of mitophagy-related genes between the two subgroups (Wilcox rank sum test). TCA, tricarboxylic acid cycle. *p < 0.05, **p < 0.01, ***p < 0.001.

To investigate the potential role of YTHDC1 in the pathogenesis of HCM, HCM patients from GSE36961 were divided into two distinct subgroups based on YTHDC1 expression (Figure 7A). Pathway enrichment analysis was conducted to explore the distinct biological features between groups. A total of 11 significantly different HALLMARKS pathways were identified, mainly associated with metabolism. In the subgroup with high YTHDC1 expression, fatty acid metabolism, glycolysis and oxidative phosphorylation were less enriched (Figure 7B). Besides, 32 different KEGG pathways were identified. Apoptosis, oxidative-phosphorylation, glycolysis-glucogenesis were less enriched in subgroup with high IGFBP3 (Figure 7C). In sum, higher YTHDC1 seemed to be consistent with less-activated material and energy metabolism.

Then, WGCNA was performed to identify the YTHDC1-related hub module in HCM. Among the six modules, the yellow one was determined to be most significantly correlated with YTHDC1 subgroups (Cor = 0.55, *p* < 0.001) (Figure 7D). The most enriched pathway from GO analysis was generation of precursor metabolites and energy, consistent with the results from GSVA (Figure 7E). Likewise, KEGG analysis draw similar results with HIF-1 signaling pathway, carbon metabolism and glycolysis-gluconeogenesis being the top three significant pathways (Figure 7F).

In order to further explore the role of YTHDC1 in energy metabolism of HCM cardiac tissues, ssGSEA was used and six pathways involving glycolysis, fatty acid β oxidation, TCA cycle and respiratory electron transport were compared between the two subgroups. The results turned out that propionyl−CoA catabolism (part of fatty acid β oxidation), TCA cycle and respiratory electron transport were less enriched in the subgroup with higher YTHDC1, consistent with the above results. Since mitochondria are the core site of energy metabolism, mitophagy was then assessed between the two subgroups. A total of 14 mitophagy-related genes were differentially expressed based on YTHDC1 expression and PINK1-PRKN mediated mitophagy was less enriched in high YTHDC1 subgroup (Figures 7G,H). These results suggested that YTHDC1 may influence mitophagy and energy metabolism in HCM cardiac tissues.

### *In vitro* experiments to verify the function of IGFBP3 and YTHDC1

**FIGURE 8:**
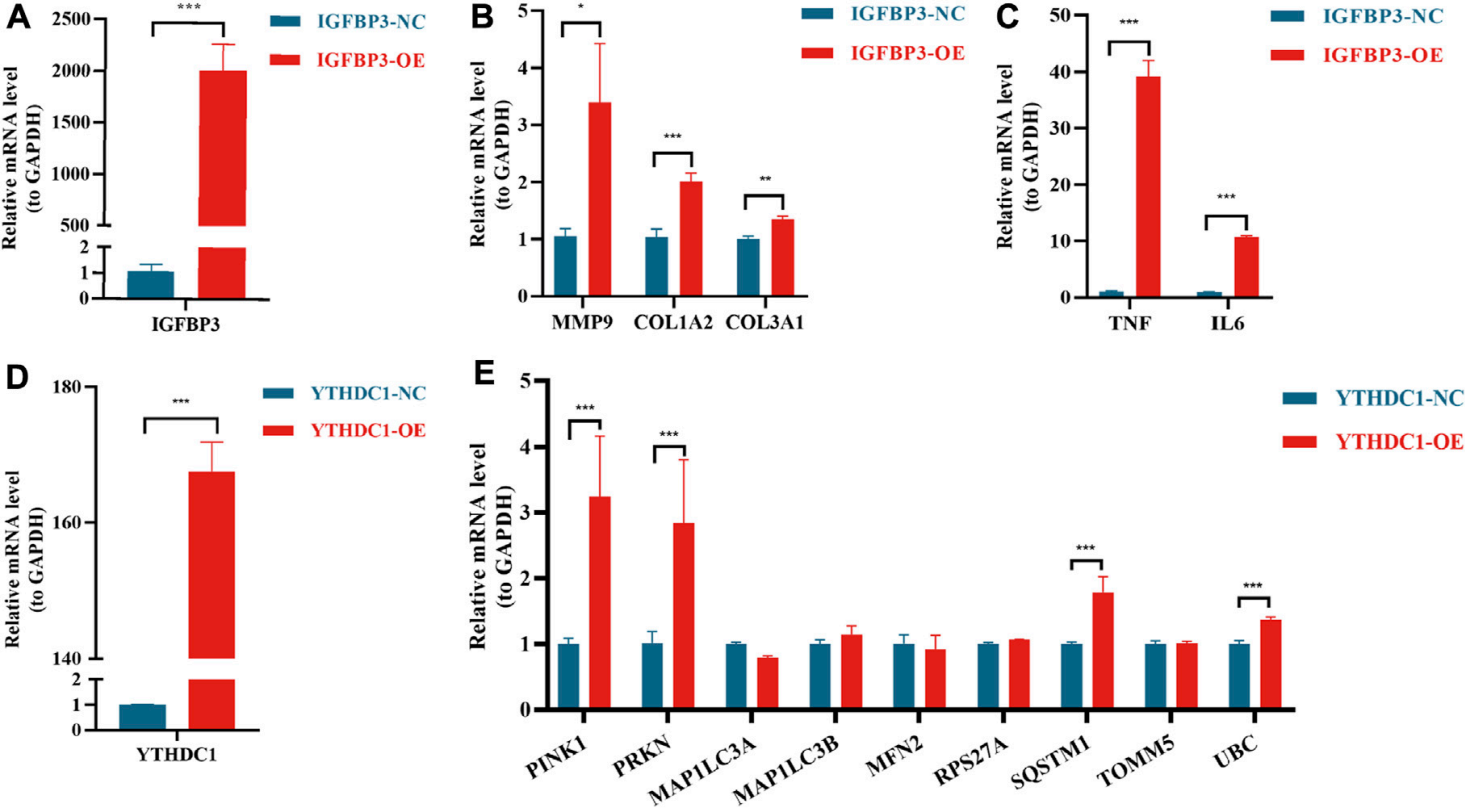
Real-time quantitative reverse transcription polymerase chain reaction analyses to verify the function of IGFBP3 and YTHDC1. **(A)** The efficiency of IGFBP3 overexpression through transfection in H9C2 cells. **(B)** Comparison of COL1A2, COL3A1 and MMP9 expression between normal control (NC) and IGFBP3 overexpression (OE) groups. **(C)** The difference in mRNA levels of TNF-α and IL-6 between NC and IGFBP3-OE groups. **(D)** The efficiency of YTHDC1 overexpression *via* transfection in H9C2 cells. **(E)** Expression of mitophagy-related genes between NC and YTHDC1-OE groups. All the above analyses were using the independent-sample *t*-test. *p < 0.05, **p < 0.01, ***p < 0.001.

In order to further verify the results from bioinformatic algorithms, overexpression of IGFBP3 and YTHDC1 in H9C2 cells was performed. IGFBP3 was successfully upregulated (Figure 8A), and the mRNA levels of ECM-related genes (Alpoim-Moreira et al., 2022), such as MMP9 (*p* < 0.05), COL1A2 (*p* < 0.001) and COL3A1 (*p* < 0.01), were significantly increased in the overexpression group (Figure 8B). Besides, inflammation-related genes TGF-α and IL-6 were also significantly upregulated (*p* < 0.001) (Figure 8C). Likewise, the mRNA level of YTHDC1 was increased by 167.6 times in transfected H9C2 cells (Figure 8C). Mitophagy-related hub genes including PINK1, PRKN, SQSTM1 and UBC were significantly upregulated consistent with the overexpression of YTHDC1 (*p* < 0.001) (Figure 8D).

## Discussion

Our study is the first to investigate the role of m6A methylation modification in the diagnosis and pathogenesis of HCM. The main findings were demonstrated as follows: 1) Significant difference existed in m6A landscape between HCM and non-HCM patients, among which YTHDC1 and IGFBP3 were identified as the novel biomarkers, both up-regulated in HCM group. 2) Two distinct subgroups based on IGFBP3 expression were established and highly infiltrated immune cells (MDSC, macrophages, etc.), more enriched immune-related pathways (TNFα signaling *via* NFκB and IL6-JAK-STAT3 signaling) and cardiac remodeling-associated pathways (epithelial mesenchymal transition, angiogenesis, etc.) were identified in the subgroup with higher IGFBP3. 3) Higher YTHDC1 expression seemed to be consistent with less-activated mitophagy (PINK1-PRKN mediated mitophagy) and energy metabolism (propionyl−CoA catabolism, TCA cycle and respiratory electron transport). 4) The overexpression of IGFBP3 could increase the mRNA level of ECM-related (COL1A2, COL3A1, MMP9) and inflammation-related genes (TNFα and IL6), while the overexpression of YTHDC1 increased the expression of mitophagy-related genes (PINK1, PRKN, etc.) in cardiomyocytes.

Currently, the diagnosis and management of HCM rely on imaging strategies (echocardiography or cardiac magnetic resonance) in combination with genetic testing for a disease-causing sarcomere mutation (Maron et al., 2022). However, heterogeneity of phenotypic expression existed among HCM patients and clinical phenotype was not always consistent with genetic features (Hathaway et al., 2021), which could cause confusion in the diagnosis and management of HCM. Discrimination of HCM in hypertension patients with LV wall thickness ranging from 13 to 18 mm is a common clinical dilemma (Maron et al., 2022). It is thus imperative to find reliable biomarkers capable of distinguishing HCM. M6A methylation modification, as the most common type of RNA modification, participates in universal biological processes (Roundtree et al., 2017a). M6A was also reported to be involved in various cardiovascular diseases, such as coronary heart disease, hypertension, and heart failure (Wu et al., 2020). However, whether m6A regulators play a role in the pathogenesis of HCM remained unknown. In the current study, distinct m6A landscape was shown between HCM and the control group, and a significant gene signature involving five m6A regulators was established to distinguish HCM with an AUC of 0.979, indicating that m6A modification may play a role in the pathogenesis of HCM. Further external validation identified IGFBP3 and YTHDC1 (both were m6A “readers”) as novel biomarkers of HCM, which may facilitate the clinical diagnosis of HCM.

IGFBP3 is the most abundant insulin-like growth factor 1 (IGF-1) binding protein, regulating proliferation, differentiation, and cell survival in various tissues (Stienen et al., 2020). For cardiac tissue, the suppression of IGFBP3 could alleviate cardiac fibrosis and cardiac remodeling in diabetic cardiomyopathy (Li et al., 2020). However, the effects of IGFBP3 on HCM were barely reported. To explore the role of upregulated IGFBP3 in the pathogenesis of HCM, two distinct subgroups were established in terms of IGFBP3 expression and functional analysis between subgroups was performed through a series of bioinformatical algorithms. The results turned out that cardiac remodeling-associated pathways seemed to be more enriched in the subgroup with high IGFBP3 expression, consistent with the role of IGFBP3 in diabetic cardiomyopathy mentioned above. Epithelial mesenchymal transition (EMT) was the most highly enriched Hallmarks pathway in the subgroup with high IGFBP3. EMT is a reversible process where epithelial cells transform into fibroblast-like cells, which not only plays an integral role in early cardiovascular development but also contributes to cardiac fibrosis in numerous cardiovascular diseases (Kalluri and Weinberg, 2009; Park et al., 2021; Usman et al., 2021). The highly enriched EMT process in high IGFBP3 subgroup suggested prominent cardiac fibrosis in this subgroup. Consistently, the GO analysis of the hub module positively correlated to high IGFBP3 drew pathways mainly associated with ECM organization. PI3K/Akt signaling pathway, the most significant pathway from KEGG analysis, was also reported to be correlated with cardiac fibrosis and cardiac hypertrophy (Weeks et al., 2017; Qin et al., 2021). By the way, the overexpression of IGFBP3 could increase the mRNA level of COL1A2, COL3A1, and MMP9, which is the main components of ECM. Therefore, IGFBP3 may promote the remodeling and fibrosis of HCM cardiac tissue.

Of note, inflammation-related pathways and immune infiltration were more enriched in the subgroup with high IGFBP3 expression, and IGFBP3 was positively correlated to the differentially infiltrated immune cells, indicating that IGFBP3 may play a role in the immune-microenvironments of HCM cardiac tissue. Immune infiltration could not only contribute to cardiac remodeling (Kologrivova et al., 2021), but also correlate to the prognosis of HCM. TNFα signaling *via* NFκB and IL6-JAK-STAT3 signaling were highly enriched in high IGFBP3 group. Consistently, *in vitro* experiments found that overexpression of IGFBP3 resulted in the upregulation of TNF-α and IL-6 in cardiomyocyte. TNF-α and IL-6 could impair diastolic function *via* downregulating the expression of sarcoplasmic reticulum Ca^2+^-ATPase channels in cardiomyocytes (Wu et al., 2011). Elevated IL-6 and TNF-α in plasma were associated with disease severity of HCM and even heart failure with preserved ejection fraction (HFpEF) (Fang et al., 2015; Markousis-Mavrogenis et al., 2019). In addition, cytokines and cytokine receptor signaling were also highly enriched in high IGFBP3 group, while PI3K/Akt signaling was the most significant pathway from KEGG analysis. It has been reported that the upregulation in PI3K/Akt signaling pathway and cytokine-cytokine receptor interactions was related to higher risks for a composite endpoint of arrhythmia, heart failure, stroke, and sudden cardiac death among HCM patients (Liang L. W. et al., 2022). In a word, high IGFBP3 expression was consistent with more activated immune and inflammation status, which may indicate worse prognosis in HCM patients.

YTHDC1, a nuclear RNA-binding protein, mediates m6A-regulated mRNA splicing (Xiao et al., 2016), nuclear transport (Roundtree et al., 2017b) and gene translation silencing (Patil et al., 2016). Few studies have reported the influence of YTHDC1 on heart. A recent study found that depletion of YTHDC1 induced dilated cardiomyopathy phenotype and significantly prolonged the relaxing time of cardiomyocytes in mice (Gao et al., 2021). In order to uncover the role of YTHDC1 in HCM, two subgroups were established based on YTHDC1 expression. GSVA indicated that higher YTHDC1 expression was consistent with less-enriched fatty acid metabolism, glycolysis, and oxidative phosphorylation. Consistently, the most enriched pathway from GO analysis of the hub module negatively related to high YTHDC1 was generation of precursor metabolites and energy. It was thus inferred that YTHDC1 was involved in the energy metabolism of HCM cardiac tissues. The comparison of metabolic pathways showed that propionyl−CoA catabolism, TCA cycle and respiratory electron transport was less-activated in high YTHDC1 subgroup. Of note, propionyl−CoA catabolism was part of the process of fatty acid β-oxidation, which was the main energy source of cardiac tissues (Wongkittichote et al., 2017). Since mitochondria are the core site of energy metabolism and activation of mitophagy pathways to eliminate aberrant mitochondria is essential for maintaining cellular homeostasis and improving mitochondrial function in cardiomyocytes (Saito and Sadoshima, 2015; Ranjbarvaziri et al., 2021), mitophagy was then assessed between the two subgroups. The result turned out that PINK1-PRKN mediated mitophagy was less enriched in high YTHDC1 subgroup. In a word, upregulation of YTHDC1, less-enriched energy metabolism and less-activated mitophagy were unified in the same subgroup. However, *in vitro* experiments showed that the overexpression of YTHDC1 could increase the mRNA level of PINK1 and PRKN, consistent with previous study demonstrating that overexpression of YTHDC1 could promote autophagy in diabetic skin (Liang D. et al., 2022). Since mitophagy insufficiency is a common pathogenic mechanism in HCM patients (Ranjbarvaziri et al., 2021), it was thus speculated that the high expression of YTHDC1 in the subgroup with less-activated mitophagy may be a result of adaptive response by cardiomyocyte in HCM in pursuit of correcting mitophagy insufficiency, although not enough to fully correct the insufficiency under pathological conditions. This is the first study demonstrating the direct relationship between the m6A writer YTHDC1 and mitophagy to date. Since the failure to upregulate mitophagy partly accounted for the damaged mitochondria and energy metabolism in HCM (Ranjbarvaziri et al., 2021), the association between YTHDC1, mitophagy and energy metabolism may provide a novel therapeutic target for HCM, although further experiments are warranted to verify this.

In summary, the current study identified two m6A regulators as novel HCM biomarkers and innovatively uncovered the potential role of IGFBP3 and YTHDC1 in regulating immune microenvironment and mitophagy of HCM cardiac tissues respectively. This finding not only provided accessory biomarkers to facilitate the clinical diagnosis, but also indicated the pathogenesis of HCM from a new perspective of m6A modification, although further researches were in need to verify this. Nevertheless, certain limitations still existed in the current study. On one hand, the research was performed using bioinformatics analysis from the transcriptomic profiles of the public database, which may be different from actual scenarios. Although external validation and *in vitro* experiments performed in the current study preliminarily justified those results, human blood samples or cardiac samples were still in need for further verification. On the other hand, the grouping method used in the current study was compromised due to the absence of clinical data, taking just the median expression of YTHDC1 and IGFBP3 as the cutoff value respectively. Although continuous distribution of the data minimized the grouping bias, it would have been better to use the value correlating to symptomatology, such as clinical severity. The correlation between gene expression and clinical manifestations remains to be further clarified. Besides, detailed prognostic information was also unavailable, which limited the further exploration on the prognosis of m6A subgroups.

## Conclusion

IGFBP3 and YTHDC1 well distinguished HCM and may facilitate clinical diagnosis. IGFBP3 may play a role in the immune-microenvironments and remodeling of cardiac tissues, while YTHDC1 may influence mitophagy and energy metabolism in HCM.

## Data Availability

The datasets presented in this study can be found in online repositories. The names of the repository/repositories and accession number(s) can be found in the article/Supplementary Material.
