# Supplementary material for: Identification and verification of IGFBP3 and YTHDC1 as biomarkers associated with immune infiltration and mitophagy in hypertrophic cardiomyopathy
Source: Front Genet. 2022 Oct 4;13:986995. doi: 10.3389/fgene.2022.986995 (PMC9577180; doi:10.3389/fgene.2022.986995)
Supplement: Supplementary file 1 [file Table1.DOCX]

Supplementary Table 1 Primer sets of targeted genes

| Gene | Sequence (5’→3’) | |
| --- | --- | --- |
|  | Forward primer | Reverse primer |
| IGFBP3 | GGAGGACCACAATGCTGGGA | TCTGGGTGTCTGTGCTCTGG |
| COL1A2 | TCCCAAAGGCAACAGTGGT | CCACGGGCTCCTCGTTTTCC |
| COL3A1 | CAGCTGGCCTTCCTCAGACTT | GCTGTTTTTGCAGTGGTATGTAATG |
| MMP9 | CACGACAGCTGACTACGACAC | GCAGGCAGAGTAGGAGTGG |
| TNF-α | GATCGGTCCCAACAAGGAGG | GCTTGGTGGTTTGCTACGAC |
| IL-6 | TCTGGTCTTCTGGAGTTCCG | AGCATTGGAAGTTGGGGTAGG |
| YTHDC1 | TTTGCAGGCGTGAATTACCC | AAGACAAAGCTGGGTTCCAC |
| PINK1 | GTGTCTGACCCACTGGACAC | CTGCTCCCTTTGAGACGACA |
| PRKN | GGCCTCCAAGGAAACCATCA | TCCACTCACAGCCACAGTTC |
| MAP1LC3A | ATCCACACCCATCGCTGAC | AGAAGCCGAAGGTTTCTTGG |
| MAP1LC3B | GAAGACCTTCAAACAGCGCC | CCAGGAGGAAGAAGGCTTGG |
| MFN2 | AGACTCATCGCCCTCTCCTT | CTTGAAGGCCCTCTCCTTGG |
| RPS27A | TCTCGTGCTGAGACTTCGTG | TGCCTGTCAAAGTGGCTACC |
| SQSTM1 | TGTACCCACATCTCCCACCA | GCCTTCATCCGAGAAACCCA |
| TOMM5 | TGTTCCGGATCGAAGGTCTC | AGACCACATCCTCACGCATC |
| UBC | ACACCAAGAAGGTCAAACAGGA | AGAACAAGCACAAGAAGGGCT |
| GADPH | CCTTCCGTGTTCCTACCCCC | TAGCCCAGGATGCCCTTTAG |
